# CBCT Evaluation of Maxillary Incisive Canal Characteristics Among Population in Regard to Possibility of Implant Cortical Anchorage—A Multicenter Study

**DOI:** 10.3390/dj13050211

**Published:** 2025-05-14

**Authors:** Fodor Calin, Bartosz Dalewski, Maciej Ellmann, Paweł Kiczmer, Stefan Ihde, Marta Bieńkowska, Jacek Kotuła, Łukasz Pałka

**Affiliations:** 1 Dr. Fodor Romulus Calin‘s Clinic of Dentistry and Implantology, Romania str. Dragos Voda nr. 8, 405300 Gherla, Romania; 2 Chair and Department of Dental Prosthetics, Pomeranian Medical University, 70-111 Szczecin, Poland; 3 ELLMED Centrum Stomatologii i Specjalistyki Ogólnomedycznej Plac Niepodległości 43, 62-035 Kórnik, Poland; 4 Private Medical Practice Paweł Kiczmer, Podgórska Street 75, 41-705 Ruda Śląska, Poland; 5 Evidence & Research Department, International Implant Foundation, Leopoldstr. 116, 80802 Munich, Germany; 6 REG-MED, Rzeszowska 2, 68-200 Żary, Poland; 7 Department of Dentofacial Orthopedics and Orthodontics, Wroclaw Medical University, Krakowska 26, 50-425 Wroclaw, Poland; j_kotula@poczta.onet.pl; 8 Private Dental Practice, Rzeszowska 2, 68-200 Żary, Poland

**Keywords:** implant placement, nasopalatine canal, NSP, CBCT, cortical anchorage

## Abstract

**Background/Objectives:** Implant placement in cases of severe bone atrophy or compromised alveolar bone requires careful planning, especially in the anterior maxilla. The nasopalatine canal (NPC) and its cortical walls offer potential anchorage sites. This study evaluates the NPC’s anatomical characteristics using cone beam computed tomography (CBCT) to assess its suitability for implant anchorage. **Methods:** A retrospective analysis of 150 CBCT scans from three dental clinics in Poland was conducted. NPC measurements—including length, width, number of canals, and distances to adjacent anatomical structures—were taken in the sagittal, coronal, and axial planes. Statistical tests included Pearson correlation and Student’s *t*-test to explore relationships between NPC dimensions and gender. **Results:** The mean NPC length was 10.27 mm and mean width 3.55 mm. Significant gender differences were observed in the canal length, width, and distances to the labial and palatal plates (*p* < 0.05). Strong positive correlations were found between the canal width at the palate base and other parameters, such as the midpoint width (r = 0.58) and diameter (r = 0.44). The distance from the palatal opening to the labial plate showed the strongest correlation (r = 0.67), indicating enhanced cortical anchorage potential with increased canal dimensions. **Discussion:** NPC morphology varied (cylindrical, funnel-like, hourglass), aligning with prior studies. Larger diameters were linked to single-canal configurations. Implant placement strategies—such as direct canal insertion or lateralization—can be effective, especially with polished, single-piece implants that reduce soft tissue ingrowth and improve primary stability. **Conclusions:** Understanding NPC anatomy is crucial for implant planning in atrophic maxillae. With the proper technique, NPC use for cortical anchorage is a viable treatment option.

## 1. Introduction

One of the first decisions for the implantologist to make is where to place an implant and how to anchorage it. If the available bone is sufficient, placing the implant intraosseous in the middle of the alveolar process to ensure axial loading is the first choice [1]. The problem is how to successfully place implants in cases of severe bone atrophy or poor alveolar bone type. Up to date, many different concepts have been proposed including cortical anchorage in the floor of the nose or the maxillary sinus, tubero-pterygoid implants, nazalus, etc., for the maxilla and mylohyoid ridge or buccal anchorage for the posterior of the mandible [2,3]. Unfortunately, since those concepts are not options on a daily basis, they require additional surgical training, experience, and profound anatomical understanding of the operating sites as in cases of implant failure and additional bone loss, there may not be another chance for implant placement.

Recently, subperiosteal implants have also regained clinicians’ interest. Especially with the development of 3D printing and digital planning, this solution has become more available and predictable for both patients and treatment providers. This allows practitioners to use subperiosteal implants not only in cases of full-arch rehabilitations but also in cases of segments, decreasing the risk of complications. What all these approaches mentioned have in common is that over time, it turned out that even with a small amount of available cortical bone, this anchorage results in such a great primary implant stability that it even allows for immediate loading of the implants [4,5]. From there, it was only a small step to resolve some of the other problems of modern implantology, i.e., a micro-gap between abutment and the implant body or unfavorable implant angulation and introducing single-piece implants [6,7]. Subsequently, this protocol forced changes in the way clinicians looked at the classic, time-dependent osseointegration concept. New definitions including bone osseofixation and corticalization emerged, triggering discussion about the role and necessity of rough surfaces in dental implantology and the way the bone reacts to foreign bodies such as dental implants [8,9,10].

Cortical anchorage means that the implant threads or tip need to be within contact of the cortical bone layer, whose thickness can vary from 1 mm to 2 mm [4,11]. Clinically, in heavily atrophied maxillae, one of the structures available for such anchorage are the cortical walls of the nasopalatine canal (NPC).

Recently, the possibilities of using the NPC or the vicinity for implant anchorage have regained popularity [12,13]. Some clinicians advocate placing implants in contact with the cortical bone of the NPC, while others suggest locating them within the canal itself, either after canal grafting or the lateralization of its content [14,15,16,17,18,19]. This canal is located in the frontal part of the hard palate in the middle line just behind the incisors. It begins with the fossa incisive and then the canal splits upwards and ends on both sides of the nasal septum in the anterior segment of the nasal cavity with incisive holes. Some authors emphasize that it should not be confused with the nasopalatine duct, which is an epithelialized structure within the NPC [20,21,22]. The NPC presents great variability in its morphology and shapes among the population [23,24,25,26]. It may have branches departing on both sides, as well as additional openings like the foramina of Scrapa (anterio-posterior of the NPC) and foramina of Stensen (medio-lateral) [27]. It contains nasopalatine vessels and nerves, branches of the maxillary division of the trigeminal nerve, and the maxillary artery [28].

The awareness of possible configurations within these anatomical structures is of the utmost importance during planning and implant placement. With the use of CBCT imaging for precise estimation of the available cortical bone, different techniques and placement methods can be used to increase the patient’s safety and clinical success of the implant treatment [29,30]. The dental implant placement in the anterior region of the maxillae, especially in the cortical bone of the incisive canal area, can improve biomechanics by decreasing the loading and bending moment created during the chewing of implant-supported prosthetics, which is crucial for long-term success [31,32]. The aim of this study was to evaluate CBCT-based evaluation NPC characteristics in regard to possible implant positioning within the anterior maxillary area.

## 2. Material and Methods

This observational study was designed as a retrospective analysis of CBCT images obtained between January 2021 and December 2022 from three dental clinics in Poland. The CBCT scans were acquired using the Pax-3D Smart Tomograph (Vatech, Hwaseong, Republic of Korea) with a basic voxel size of 0.2 mm, exposure settings of 8.4 mA and 94 kV, and a scanning time of 18 s. The cylindrical fields of view measured 100 × 80 mm. Images were reconstructed with a slice thickness of 0.2 mm at 0.2 mm intervals.

The dataset included 150 CBCT scans of the anterior maxilla region, selected retrospectively from patient archives. Inclusion was limited to cases with complete visualization of the maxillary bone from the alveolar crest to the pyriform aperture. Participants were not actively recruited, and CBCT images were anonymized prior to analysis, retaining only demographic data (age and gender). Therefore, patient consent was not required, as scans had been originally acquired for clinical purposes unrelated to this study.

To ensure objectivity and minimize selection bias, CBCT scans were randomly selected using a computer-generated randomization algorithm from the anonymized pool of eligible cases that met the inclusion criteria. No identifying information was accessible to the researchers at any point in the selection or analysis process.

**Figure 1:**
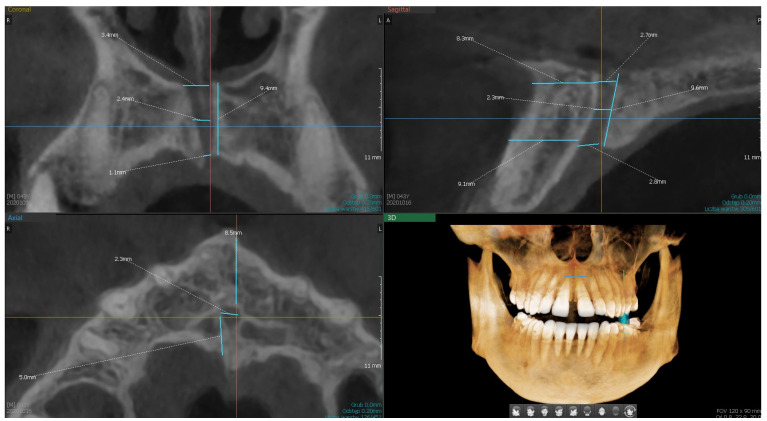
CBCT images for assessment and measurements of the regional morphology, available bone, and accessory canals in three radiological sections (axial, sagittal, coronal).

Assessment and measurements of anatomical structures, including regional morphology, bone availability, and presence of accessory canals, were performed independently by two experienced implantologists. These evaluations were conducted on three radiological planes (axial, sagittal, coronal) using specialized imaging software (ver.1.0.6.0.1, Ez3D-i; Vatech, Republic of Korea) (Figure 1). To verify intra- and inter-observer reliability, a subset of the scans was randomly re-evaluated after several weeks. Additionally, two independent researchers reviewed the images to confirm measurement accuracy and consistency. Discrepancies were resolved through discussion and consensus.

To reduce potential observer bias, the evaluators were blinded to patient demographic data during image assessment. Furthermore, the study adhered to the Strengthening the Reporting of Observational Studies in Epidemiology (STROBE) guidelines and was conducted in accordance with the Declaration of Helsinki.

Statistical analysis was performed to evaluate the morphological characteristics of the nasopalatine canal (NPC). Descriptive statistics were calculated for all variables, and Pearson correlation coefficients were used to assess linear relationships between anatomical parameters and demographic variables. Significance was set at *p* < 0.05.

Regarding the measurements of NPC features and the distance of the NPC to adjacent anatomical structures, the following dimensions were assessed:

Measurements in the “sagittal” plane:

S1.—length of the canal from the base of the nose to the palate;

S2.—width of the canal at the base of the palate;

S3.—width of the canal at the base of the nose;

S4.—width of the canal in half-length;

S5.—distance to the labial plate at the level of the mouth opening of the palatine canal;

S6.—distance to the labial plate at the level of the nasal outlet.

Measurements in the “coronal” plane:

C1.—width of the canal at the base of the nose;

C2.—width of the canal in half;

C3.—width of the canal at the base of the palate;

C4.—canal length;

C5.—number of canals.

Measurements in the “axial” plane:

A1.—number of openings;

A2.—diameter of the main canal;

A3.—distance to the palatal plate (bone thickness);

A4.—distance to the labial plate.

## 3. Results

A statistical analysis was conducted on the dataset containing measurements of NPC characteristics (Figure 1). The analysis includes the variables presented above. Out of the examined 150 CBCT scans, for variables S3, S6, and C2, 1 radiological examination each was excluded due to the inability to be measured. For each variable, basic statistical measures such as the mean, median, minimum, maximum, quartiles, and standard deviation were calculated. The results of the statistical analysis are presented in Table 1.

The Pearson correlation analysis was conducted between NPC characteristics. Pearson correlation (r) measures the strength and direction of a linear relationship between two quantitative variables. The correlation value ranges from −1 to 1, where r = 1 indicates a perfect positive correlation, r = −1 indicates a perfect negative correlation, r = 0 indicates no linear correlation between the variables. For medical sciences, the following ranges of absolute values of the Pearson correlation coefficient, used for interpreting the obtained results, are applied:

<0.2 weak correlation;

0.3–0.5 fair correlation;

0.6–0.7 moderate correlation;

0.8–0.9 very strong correlation;

1 perfect correlation.

For the length of the canal from the base of the nose to the palate (S1), a weak positive correlation was found with the variable S5 (r = 0.19), while a fair positive correlation occurs between S1 and S6 (r = 0.31) as well as S1 and A3 (r = 0.23). S1 is most strongly correlated with the variable C4, which is a moderate positive correlation (r = 0.58). One could assume that with age, the length of the canal from the base of the nose to the palate decreases (the correlation between S1 and age is r = −0.17); however, this represents a weak negative correlation.

With the increase in the width of the palate base (S2), the following parameters increase: C4 (r = 0.18), with a weak correlation; S3 (r = 0.37), C1 (r = 0.32), C2 (r = 0.44), C3 (r = 0.46), and A2 (r = 0.44)—these parameters have a fair correlation; and S4 (r = 0.58), which is a correlation of moderate strength. On the other hand, a negative correlation is observed for parameter S2 with S5 (r = −0.21), C5 (r = −0.30), and A1 (r = −0.24) and these correlations are fair.

The width at the base of the nose (S3) is fairly positively correlated with variables S2 (r = 0.37), S4 (r = 0.53), C1 (r = 0.44), C2 (r = 0.41), C3 (r = 0.35), and A2 (r = 0.39), while a weak negative correlation is observed between parameter S3 and S6 (r = −0.23).

For parameter S4 (width at the midpoint), there is a moderately positive correlation with variables S2 (r = 0.58), S3 (r = 0.54), C2 (r = 0.57), and A2 (r = 0.57), a fair correlation with variables C1 (r = 0.43) and C3 (r = 0.30), and a weak correlation with age (r = 0.18). Negative correlations for parameter S4 are observed with S5 (r = −0.32), S6 (r = −0.23), C5 (r = −0.21), and A4 (r = −0.30), which are fair correlations.

The strongest positive correlation (r = 0.67) in the examined dataset is observed between variable S5 (distance to the lip plate at the level of the palatal canal opening) and variable A4 (distance from the lip plate). An increase in one distance is associated with an increase in the other. Additionally, variable S5 has a positive correlation with parameter S6 (r = 0.50), C4 (r = 0.26), C5 (r = 0.17), and A3 (r = 0.32). However, there is also a negative correlation with variables C2 (r = −0.18) and A2 (−0.32).

The distance to the lip plate at the level of the nasal canal opening (S6), apart from dependencies within the parameters S, has only a positive correlation with variable A4 (r = 0.53), and a negative correlation with A2 (−0.27).

For measurements in the “coronal” plane, in addition to the correlations mentioned above, there are also the following positive correlations of moderate strength: C1 and C2 (r = 0.54), C1 and A2 (r = 0.54), C2 and A2 (r = 0.64), and C5 and A1 (r = 0.67). A slightly smaller direct relationship was observed between the following variables: C1 and C3 (r = 0.36), C1 and C4 (r = 0.19), C2 and C3 (r = 0.47), C2 and age (0.17), C3 and C4 (r = 0.37), C3 and A2 (r = 0.40), C4 and A2 (r = 0.26), and C4 and A4 (r = 0.16). Weak negative correlations occur between variables C2 and A4 (r = −0.20), C3 and A1 (r = −0.19), and C4 and age (r = −0.17) as well as C5 and age (r = −0.17).

Apart from the correlations mentioned above, variables for measurements in the “axial” plane exhibit a weak positive correlation between variables A3 and A4 (r = 0.22), and between A2 and age (r = 0.17). Additionally, there is a negative correlation of fair strength between A2 and A4 (r = −0.34).

## 4. Discussion

When assessing the NPC in the sagittal plane, we can distinguish five shapes as per the literature: cylindrical—with the highest prevalence of more than 50%, cone—characteristic for small-diameter NPC (<3 mm), funnel-like—mostly found in severely resorbed ridges, and hourglass and banana-like—encountered in 14.5–38.8% and 3.9–16.0% cases, respectively [33,34,35]. Interesting variabilities in the canal numbers of the NPC can be found in the frontal view of the radiological examination. As the Y-shaped two NPCs seem to be most prevalent, they may have an additional two, three, or even four canals; some studies like Etoz and Sisman [36] report the prevalence of a single NPC in 96.1% with one nasal opening in 44.3%. Additionally, Bornstein et al. [37] found a relationship between the incisive canal mean diameter and type of configuration with single canals presenting the largest mean diameter (4.45 mm). Taking into consideration the differences in the study design based on the available literature, the lengths of the NPC ranged from 8.1–15.1 mm, the width of the canal was 2.0–4.3 mm, and the widths of the openings were 2.4 mm (palatal opening) and 4.9 mm (nasal cavity opening) [28]. According to Güncü et al. and Acar and Kamburoglu, males have longer canals than females [24,38]. What is interesting is the absence of teeth in the anterior maxilla decreased the NPC length, but the canal diameter remained unchanged [24,34].

The concept of utilizing the nasopalatine canal for implant anchorage is not new [39]. Over the years, different authors have suggested direct implant placement in the canal after the excavation of its content with a bur or curette, and the lateralization of the canal structure with its subsequent augmentation with a bone chip or graft material [40,41,42,43]. These treatment options were made for classic, rough-surface endosseous implants; thus, they share their limitations like a maximum implant diameter of 5.5–6 mm and the need to direct bone implant contact to prevent soft tissue ingrowth [44,45,46]. According to the literature, possible complications that may occur regarding implant placement in the NPC result from the content of the nasopalatine canal and include bleeding, sensory disturbance, and lack of osseointegration [19,35,43,47].

Other available options include single-piece screw implants with a polished surface. The minimal diameter of those implants is 2.7Ø mm and the maximum is 12Ø mm, which covers the maximum NPC diameter presented in our study. In cases of immediate loading protocol, utilizing the cortical walls of the nasopalatine canal is enough to achieve primary stabilization by osteofixation; thus, a rough surface is not required. Depending on the clinical situation, the implant can be placed directly into the NPC, crossed in the anterio-posterior or mesio-distal direction, or be in contact with one of the NPC walls. The results obtained in this study show that in a heavily atrophied maxillary crestal ridge, utilization of the NPC for implant anchorage is one of the possible treatment options after the careful selection of the implant type.

The measured length of the canal (C4 = 10.27 mm) is slightly below the mean reported in most studies, most of which ranges between 10.9 and 16.5 mm. The results of this study are consistent with those presented by Liang et al. (9.9 mm) and Mraiwa et al. (10.9 mm) [23,46]. The results are within the normal anatomical variation, though they sit on the shorter end of the spectrum.

The mean width of the nasopalatine canal in the base of the nose (4.06 mm) is slightly wider than average; most studies report values between 3.2 and 3.8 mm [37]. The width in the middle of the canal (3.32 mm) falls well within the range of previously reported values (2.6–3.5 mm) [37,46]. The base of the palate with a width of 3.87 mm matches or slightly exceeds the average values from multiple studies [37,46].

### 4.1. Limitations

Despite offering valuable insights into the anatomical characteristics of the nasopalatine canal (NPC) for implant planning, this study presents several limitations. Firstly, the retrospective design restricts control over variables such as patient selection and scan quality, potentially introducing selection bias. The absence of clinical follow-up or outcome data limits the ability to correlate anatomical measurements with implant success or complication rates. Additionally, the study’s population, drawn solely from three clinics in Poland, may not represent broader demographic or ethnic variations, thereby reducing generalizability. While the analysis provides detailed anatomical measurements, it does not address critical factors such as bone density or neurovascular risks, which are essential for comprehensive implant planning. Furthermore, although multiple evaluators assessed the scans, the lack of reported inter-observer reliability introduces the possibility of measurement bias. Finally, while several statistically significant correlations were identified, their clinical relevance remains uncertain and warrants further investigation in prospective, outcome-based studies.

### 4.2. Future Scope

This study highlights the evolving perspective on the nasopalatine canal (NPC) and its surrounding anatomical structures as potential sites for strategic implant anchorage, particularly in the context of atrophic maxillae. Once considered a limiting factor in implant placement, the NPC is now gaining recognition for its surgical relevance, thanks to advancements in three-dimensional imaging and a better understanding of maxillary bone anatomy.

Through precise CBCT-based evaluations, this investigation demonstrates the variability and potential of the premaxillary cortical bone surrounding the NPC. The findings support the notion that with careful planning, the NPC region can serve as a viable alternative to more invasive bone augmentation techniques. Such minimally invasive approaches not only preserve patient anatomy but also reduce treatment time, morbidity, and costs.

While these insights are promising, they underscore the importance of individualized treatment planning and a cautious, evidence-based approach to novel implant anchorage concepts. Continued research, both in vitro and in vivo, is essential to validate the biomechanical feasibility, long-term success, and safety of implants involving the NPC region.

Reimagining the anatomical boundaries of implant placement opens new horizons in maxillofacial rehabilitation—especially for patients with severe bone loss—marking a progressive shift toward more personalized, anatomy-driven implantology.

## 5. Conclusions

To fully utilize the NPC for implant anchorage, clinicians must be aware of its anatomical variations. The findings of this study indicate that in cases of severe maxillary atrophy, the NPC can serve as a viable implant anchorage site when the appropriate implant type and diameter are carefully selected.

## Figures and Tables

**Table 1:** Statistical analysis for each variable.

Variable	Descriptive Statistics							
	Valid N	Mean	Median	Min	Max	Q1	Q3	SD
S1.—length of the canal from the base of the nose to the palate	150	9.67	9.60	4.00	16.60	8.00	11,20	2.47
S2.—width of the canal at the base of the palate	150	3.71	3.55	1.10	7.60	2.90	4.50	1.28
S3.—width of the canal at the base of the nose	149	3.30	3.00	0.90	6.90	2.30	4.20	1.29
S4.—width of the canal in half-length	150	2.62	2.50	0.80	6.70	1.60	3.30	1.14
S5.—distance to the labial plate at the level of the mouth opening of the palatine canal	150	6.66	6.75	2.00	11.30	5.70	7.70	1.73
S6.—distance to the labial plate at the level of the nasal outlet	149	9.31	9.10	4.80	14.40	8.00	10.90	2.15
C1.—width of the canal at the base of the nose	150	4.06	4.15	1.20	9.90	3.00	5.00	1.38
C2.—width of the canal in half	150	3.32	3.25	1.20	6.30	2.40	4.00	1.16
C3.—width of the canal at the base of the palate	150	3.87	3.70	1.10	9.20	2.60	5.00	1.54
C4.—canal length	150	10.27	10.10	1.00	21.10	7.70	12.70	3.52
C5.—number of canals	150	1.15	1.00	1.00	2.00	1.00	1.00	0.36
A1.—number of openings	150	1.09	1.00	1.00	3.00	1.00	1.00	0.31
A2.—diameter of the main canal	149	3.55	3.40	1.00	9.00	2.50	4.30	1.41
A3.—distance to the palatal plate (bone thickness)	150	3.57	3.30	0.60	7.80	2.40	4.70	1.55
A4.—distance to the labial plate	150	6.49	6.45	1.40	10.40	5.40	7.50	1.78
age	150	51.65	52.00	18.00	81.00	43.00	62.00	14.16

## Data Availability

The data presented in this study are available on reasonable request, after the signature of a formal data sharing agreement in anonymous form, from the corresponding author because they are protected by privacy.
